# Tracking the timely dissemination of clinical studies. Characteristics and impact of 10 tracking variables

**DOI:** 10.12688/f1000research.17022.1

**Published:** 2018-11-29

**Authors:** Daniel Strech, Sören Sievers, Stefanie Märschenz, Nico Riedel, Susanne Wieschowski, Jörg Meerpohl, Holger Langhof, Stephanie Müller-Ohlraun, Ulrich Dirnagl

**Affiliations:** 1 QUEST Center, Berlin Institute of Health (BIH), Berlin, Germany; 2 Charité - Universitätsmedizin Berlin, Berlin, Germany; 3 Institute for History, Ethics and Philosophy of Medicine, Hannover Medical School, Hannover, Germany; 4 NeuroCure Clinical Research Center, Charité - Universitätsmedizin Berlin, Berlin, Germany; 5 Institute for Evidence in Medicine (for Cochrane Germany Foundation), Medical Center – University of Freiburg, Faculty of Medicine, Freiburg, Germany

**Keywords:** clinical studies, trials, registries, follow-up, trial tracking, university medical centers, private companies

## Abstract

**Background:** Several meta-research studies and benchmarking activities have assessed how comprehensively and timely, academic institutions and private companies publish their clinical studies. These current “clinical trial tracking” activities differ substantially in how they sample relevant studies, and how they follow up on their publication.

**Methods:** To allow informed policy and decision making on future publication assessment and benchmarking of institutions and companies, this paper outlines and discusses 10 variables that influence the tracking of timely publications. Tracking variables were initially selected by experts and by the authors through discussion. To validate the completeness of our set of variables, we conducted i) an explorative review of tracking studies and ii) an explorative tracking of registered clinical trials of three leading German university medical centres.

**Results:** We identified the following 10 relevant variables impacting the tracking of clinical studies: 1) responsibility for clinical studies, 2) type and characteristics of clinical studies, 3) status of clinical studies, 4) source for sampling, 5) timing of registration, 6) determination of completion date, 7) timeliness of dissemination, 8) format of dissemination, 9) source for tracking, and 10) inter-rater reliability. Based on the description of these tracking variables and their influence, we discuss which variables could serve in what ways as a standard assessment of “timely publication”.

**Conclusions:** To facilitate the tracking and consequent benchmarking of how often and how timely academic institutions and private companies publish clinical study results, we have two core recommendations. First, the improvement in the link between registration and publication, for example via institutional policies for academic institutions and private companies. Second, the comprehensive and transparent reporting of tracking studies according to the 10 variables presented in this paper.

## List of abbreviations

CD, completion date; CENTRAL, Cochrane Central Register of Controlled Trials; CSR, clinical study reports; EU, European Union; FDA, Food and Drug Administration; FDAAA, Food and Drug Administration Amendments Act; GCP, Good Clinical Practice; ICH, International Council for Harmonisation of Technical Requirements for Pharmaceuticals for Human Use; ICMJE, International Committee of Medical Journal Editors; NCT, national clinical trial; PCD, primary completion date; UMC, University medical center

## Background

The results of clinical trials and observational studies form the basis of evidence-based decision making in health care, coverage decisions, the planning and funding of new research studies, ethics reviews, and research quality assessment ^1^. Over the past three decades, several meta-research projects have demonstrated that the results of clinical studies are often not reported at all, or the reporting is incomplete, biased, or inconsistent with what was planned at the protocol stage ^2– 5^.

Recent studies have compared the proportion of trials/studies with published/disseminated results across academic institutions and for-profit companies. For instance, Chen *et al*. compared the publication rates of completed trials conducted by 51 academic medical centres across the US ^6^. They found that overall, the results were published for 67% of 4,347 trials; however, the results of only 36% of trials were published within two years of study completion. They identified wide variation in the proportion of trials being published in a timely manner across the 51 medical centres. The TrialsTracker project, which started in 2016, automatically identifies completed trials on Clinicaltrials.gov, and links them with results from automated searches for published results to present data on publication rates at the individual study centre level (see TrialsTracker). In the newest version of TrialsTracker, approximately 65% of all applicable trials were found to be reported (accessed 21 March 2018). A third approach to this line of research is the “Good Pharma Scorecard”, which takes Food and Drug Administration (FDA)-approved drugs as a starting point, and provides benchmarks for transparency in industry-run clinical drug research and development ^7, 8^. In this initiative, the authors combined data from various sources (e.g., Clinicaltrials.gov, PubMed, Google Scholar) and different forms of results dissemination (summary reports, clinical study reports, peer-reviewed publications). This study revealed that of 505 trials relating to 31 drugs approved by the FDA in 2014, the results for a median of 68% of trials per drug were publicly available. The authors also found that in 233 of the 505 trials that were conducted with patients (in contrast to healthy participants), a median of 96% of trials, per drug, were publicly available.

The above-mentioned studies differed in many ways regarding their methods for sampling relevant clinical studies, and following up on their publication. What are the definition and operationalization of a “completed trial”? Should publication follow-up be applied only for prospectively registered trials, or also for trials that were registered many months or years after the study started? What databases are searched for publications? What publication formats and contents count as a “publication”? What amount of time to publication is considered “timely”? Should only clinical trials or also observational studies be followed up on? Should only completed studies or also discontinued ones be followed up on? Different decisions on the various tracking variables and decisions on other methodologically relevant issues lead to different results for how academic institutions and for-profit companies perform overall and on comparative rankings or benchmarks.

The comprehensive and timely publication of clinical study results affects the reputation of academic institutions and private companies. Such data impact public trust and the willingness of foundations to fund research. To provide a basis for informed policy and decision making through publication assessment and benchmarking, this paper aims to identify and characterize the different variables affecting the results of tracking whether and how timely results from clinical studies are published.

## Methods

The selection of tracking variables presented in this paper was initially driven by expert knowledge and discussions within the group of authors. To validate the completeness of our set of tracking variables, we then conducted i) an explorative review of studies that followed up on the publication of clinical studies and ii) an explorative follow-up study of registered clinical trials of three leading German university medical centres (UMCs): Berlin, Freiburg, and Hannover.

The explorative review started with a set of eight follow-up studies that we were aware of ^6, 8– 14^. All references of these (and the later-included) studies were evaluated for additional follow-up studies. Altogether, we identified 34 follow-up studies. All identified studies were read in full to extract reported methods for sampling and follow-up. The extracted content was checked for methodological details that our initial expert-driven set of tracking variables did not contain, and these were added to our set accordingly. The detailed results of this review of follow-up studies will be published elsewhere.

The methods for our explorative follow-up study have been published as a preregistered study protocol (Extended data ^15^). We used an R script (see Software availability) to combine all relevant datasets from clinicaltrials.gov and search criteria needed to retrieve clinical trials from all 36 German UMCs and to extract their study characteristics. For each of the included studies, a results publication was searched independently by two researchers in a 3-step process in i) the registry, ii) in Pubmed, and iii) in Google Scholar. In the meantime, we conducted our study based on this protocol, evaluating all 36 German UMC as specified by the German Medical Faculty Association (MFT). The results of this comprehensive follow-up study will be published elsewhere. Our explorative follow-up study helped to further clarify how the tracking variables described in this paper influence the results of follow-up studies.

## Results

Based on the above-mentioned expert discussion, as well as the review of existing follow-up studies, and the insights from our explorative follow-up study of three German UMC, we were able to distinguish 10 variables influencing the design and results of follow-up studies of clinical studies. Table 1 categorizes the 10 variables into two broad areas: “sampling of studies” and “follow-up of studies”.

**Table 1.** Sampling and tracking variables and related questions.

Sampling	Tracking
**1. Responsible party for clinical studies**: Should the sampling refer to the legal or ethical perspective on “reponsible party”? **2. Type and characteristics of clinical studies**: What study type (clinical trial, observational) with what further study characteristics to follow up? **3. Status of clinical studies**: Should only completed studies or also discontinued, terminated trials be followed up on? **4. Sources for sampling**: What source (registry, IRB archives etc.) should be used for identifying institution-specific clinical studies? **5. Timing of registration**: How should retrospectively registered studies be dealt with?	**6. Determination of completion date**: How should “trial completion” be defined **7. Timeliness of dissemination**: What time frames between end of trial and dissemination are specified for the follow-up? **8. Format of publication/dissemination**: What publication formats and contents should count as results publication? **9. Sources for tracking**: Where (registry, database, web engines etc.) and how should publications be searched for? **10. Inter-rater reliability**: How are interpretive judgments and inter-rater differences dealt with?

Below, we describe the tracking variables, potential challenges in their operationalization, and their current influence on publication assessments. In the Discussion section, conceptual and normative issues for all 10 variables will be explored, and we will provide practical recommendations for future study tracking.

### 1. Responsible party for clinical studies

From a legal perspective, a specific university and its clinical study investigators are responsible for the dissemination of study results only if they are the “responsible party”, that is, if they are either the sponsor and/or the principal investigator. The International Council for Harmonisation of Technical Requirements for Pharmaceuticals for Human Use (ICH) Guideline for Good Clinical Practice (GCP), for example, defines in section 1.53 that the sponsor is “an individual, company, institution, or organization which takes responsibility for the initiation, management, and/or financing of a clinical trial”. The new European Union (EU) clinical trials regulation 536/2014, which is expected to come into force in 2019, also refers to the ICH GCP. According to that legal perspective, a specific university and its investigators who are “only” cooperating as a recruiting site are “not responsible” for whether and how timely the trial results are published. From an ethical perspective, however, physician investigators should only recruit participants for clinical trials that generate social value. Every recruiting physician investigator should therefore feel responsible for facilitating the timely publication of study results.

Several follow-up studies explicitly sampled clinical trials according to this legal definition of responsibility ^6^. Other follow-up studies sampled all trials approved by local ethics committees, but did not further specify the issue of responsibility ^10, 12^. Our explorative study demonstrated that only one third of trials conducted at the three German UMC are trials for which the university is the “legally” responsible party.

### 2. Type and characteristics of clinical studies

Clinical studies are broadly differentiated into *interventional* trials and *observational* studies, with different legal requirements. Therefore, studies following up on clinical studies for a specific university must decide whether to include both or only one study type or only subgroups, such as prospective cohort studies. ClinicalTrials.gov currently (21 July 2018) lists 221,251 interventional trials and 55,875 observational studies.

Most follow-up studies we reviewed focused on interventional trials. One exception is the study by Ross *et al*. that followed up on a sample of both types of clinical studies that were registered in ClinicalTrials.gov and found that the publication rate was 56% for interventional, placebo-controlled trials and 42% for observational studies ^11^. In a recent study, Spelsberg *et al*. were able to follow up on a full sample of 558 observational post-marketing studies on adverse drug reactions ^16^. They could not find any results reported for the 558 studies in the drug regulator’s public adverse drug reactions database. A peer-reviewed journal publication was found for five (1%) post-marketing studies ^16^.

Another relevant characteristic of clinical studies is whether they are subject to *mandatory reporting* requirements. Several more recent follow-up studies excluded all trials that do not fall within the mandatory reporting rules according to the Food and Drug Administration Amendments Act (FDAAA) 801 ^7, 11, 14, 17^.

### 3. Status of clinical studies

Most follow-up studies focus on the analysis of how timely *completed* studies are published, e.g., ^11, 17, 18^. These follow-up studies therefore must exclude all *terminated*, *discontinued*, and *withdrawn* trials. Knowledge, however, is also gained from discontinued trials that, for example, report recruitment barriers or unanticipated adverse effects. A follow-up study by Pica *et al*. explicitly included all clinical trials, irrespective of whether they were completed, terminated, withdrawn, or suspended ^13^. Anderson *et al*. also included both completed and terminated studies ^14^. Others further specified the exclusion of non-completed studies. Miller *et al*., for example, stated that they excluded any trials that were terminated without participant enrolment ^7^, but they do not explicitly state how they addressed other types of terminated, withdrawn, or suspended trials. Kasenda *et al*. showed that for 1,017 clinical trials approved at one of six institutional review boards (IRBs) in Germany, Switzerland and Canada, 25% were discontinued ^12^. Discontinued trials were more likely to remain unpublished than completed trials (55% vs 34%).

### 4. Source for sampling

IRB archives should be the most sensitive and least biased source for sampling all clinical studies conducted at one specific university, at least in countries where ethics reviews of clinical studies became mandatory sometime after the 1975 Tokyo revision of the Declaration of Helsinki ^19^. The previously mentioned study by Kasenda *et al*., for example, found that 10 years after the IRB approval of 1,017 clinical trials, the results of 56% (n=567) were published as a full journal article ^12^.

In practice, however, most newer studies following up on clinical trials refer to public registries, as this is more convenient and practicable. Registries, however, rarely include all clinical studies conducted at any given university. The prospective registration of clinical trials in a public registry became legally binding under FDAAA 801 in the USA for all phase II/III trials in 2007. Another strong incentive to register clinical trials was the International Committee of Medical Journal Editors (ICMJE) policy issued in 2015, which recommends that all medical journals require prospective registration as a condition of consideration for publication ^20^. In the EU, prospective registration for clinical trials in the public EU Clinical Trials Database will become a legal requirement with EU regulation 536/2014 (Article 67). However, the same Article 67 still allows certain pieces of information not to be published, i.a. also recognizing the “legitimate economic interests” of sponsors. All clinical studies that do not fall under these laws (observational studies, trials on psychotherapy, surgery studies) can nevertheless be registered, even retrospectively. Van den Bogert *et al*. found publication rates of 75% for prospectively registered IRB-approved clinical trials and 48% for non-registered trials ^21^.

### 5. Timing of registration

The inclusion of all registered trials by studies following up on the publication of results might overestimate publication rates for another reason. Even trials with mandatory registration are sometimes registered after the sponsor plans to publish the results, and then realizes that this requires registration. Although the above-mentioned ICMJE policy explicitly refers to prospective registration, many journals also allow retrospective registration ^22, 23^.

How to determine the cut-off between prospective and retrospective registration is complicated by two points. First, legal obligations for registration allow a time window. The FDAAA 801, for example, requires trial registration within 21 days after the enrolment of the first participant. Second, several “timings” for registration can be distinguished. The ISRCTN registry, for example, reports both the date that researchers submitted their request for registration and the date that trial registration was completed.

Recent follow-up studies have differed in how they deal with retrospectively registered trials and how they define “retrospective”. Pica *et al*., for example, excluded all trials that were registered more than 60 days after the study started ^13^. Others, such as Chen *et al*. and TrialsTracker, did not exclude retrospectively registered trials at all and did not allow for subgroup analyses ^6, 24^.

In our explorative follow-up study, we found that the proportion of trials with published results was 68% for all prospectively registered trials, 73% for trials registered more than 60 days after the trial started, and 82% for trials registered after study completion. We also found 16 trials registered after the date of the first results publication, which of course have a 100% publication rate.

### 6. Determination of completion date

One can broadly distinguish three ways to determine the completion date of an interventional trial: A) the “primary completion date” (PCD), which, according to ClinicalTrials.gov, is the “date on which the last participant in a clinical study was examined or received an intervention to collect final data for the primary outcome measure”, B) the later “completion date” (CD), which is defined as the date of the last participant’s last visit to collect final data, and thus also includes secondary outcome measures and adverse events, or C) the “estimated” study completion date, which is the study completion date expected by the researchers. According to the ClinicalTrials.gov glossary, the “estimated” date is perceived in the same way as the CD.

At present, follow-up studies using registries are heterogeneous with regard to the CD definition to which they refer. Some refer to the PCD ^14, 18^, some refer to the CD ^11^, and some do not specify ^25^. A follow-up study that sampled clinical study protocols archived at IRBs regarded a study as completed if the data collection was terminated or if the study results were published ^10^.

In our pilot follow-up study, we found, unsurprisingly, that the proportion of published trials is higher for all three German UMC 24 months after the CD (35% of trials published) than 24 months after the PCD (28% published).

### 7. Timeliness of publication

Of course, the proportion of published studies also varies considerably with regard to what one accepts as “timely”. Both the FDA and EU legal frameworks allow 12 months for the publication of “summary results”, which form part of the respective registry entries. The FDAAA has mandated results reporting since 2008 ^26^. The EU Commission introduced similar requirements in 2014 but is still facing implementation barriers ^27^. However, no offical standards exist for how timely more detailed and contexualized results should be published in peer-reviewed journals.

Ongoing and past follow-up studies have dealt differently with how and where to set adequate time frames. TrialsTracker reports the proportion of trials for each sponsor published within 24 months after the PCD ^9, 24^. Ross *et al*. reported the publication rates for a timeframe of 30 months after the CD. Chen *et al*. focused their results reporting on 24 months after the PCD but also illustrated how the publication ratio changed with alternative timeframes ^6^. Miller *et al*. compared the publication ratio at FDA approval, and 3 months and 6 months post-approval ^8^. Many other follow-up studies have assessed the all-time publication ratio, often allowing more than 10 years between the completion and publication of the trial ^10, 12^.

In our pilot study with three German UMC, we found publication rates (including summary results) of 16% for 12 months after the CD, 35% for 24 months after the CD, and 71% for all-time follow-up.

### 8. Format of publication/dissemination

What should count as a relevant publication or other dissemination format? Peer-reviewed publications are the typical dissemination format and were accordingly accepted by almost all of the above-mentioned follow-up studies. Another newer, but increasingly accepted, dissemination format is that of the previously mentioned summary results ^6, 9^. Miller *et al*. further included clinical study reports (CSRs) ^8^. Other formats such as theses, conference proceedings, books, or data uploaded on data-sharing platforms might also reveal important and sufficient information on trial results. The 34 follow-up studies we are aware of did not include any of the latter publication formats in their searches.

Irrespective of the dissemination format, one might count only those result publications that report the essential information needed. Guidance exists on the essential content to be included in summary results (see ClinicalTrials.gov ^28^ definitions), journal publications ^29^, and CSRs ^30^. However, appraising each identified publication for its comprehensiveness and appropriateness requires considerable time. Some follow-up studies invested this time. Kasenda *et al*., for example, checked all full texts of retrieved publications and demonstrated that they often do not report on all predefined outcomes or deviate in other ways from registered information on the study design ^31^.

### 9. Sources for tracking

Time to publication also strongly depends on where publications are sought. A first obvious search can be performed on the registry itself. The ClinicalTrials.gov database highlights for each trial the “first results received”, if available. However, the “first results received” only illustrate when summary results for the trial were reported. The registry entry for each trial further automatically indexes all publications listed in PubMed that mention the trial-specific NCT identifier in the abstract (see NIH page of registry numbers) ^32^. Furthermore, ClinicalTrials.gov allows sponsors and principal investigators to manually index publications for registered trials.

Another obvious search strategy is to search PubMed for the NCT number. As the automatic indexing at ClinicalTrials.gov does not work in all cases, this approach might reveal additional result publications. In our explorative follow-up study of German UMC, we identified an additional 3% of result publications via this approach. Chen *et al*. and TrialsTracker also checked for NCT identifiers in PubMed ^6, 24^.

Further search strategies become more time intensive and require more interpretive judgements. Bluemle *et al*. searched the Cochrane Central Register of Controlled Trials (CENTRAL) ^10^. They contacted the applicants of all included protocols by personal letter. For each submitted protocol, they asked individually about the current project status, the verification of already-identified publications, and references of additional publications they may have missed. Their literature searches identified 138 full publications, and the survey identified an additional 72. However, Bluemle *et al*. did not report how much these additional publications changed the overall publication rate, which turned out to be 48% ^10^.

None of the 34 follow-up studies we are aware of searched publications for trials in general search engines such as Google, Bing, or Yahoo. In our follow-up of trials from three German UMC, we identified result publications for 48% of all trials by searching summary results and indexed publications at the respective registry entries, combined with PubMed searches for NCT identifiers. However, additional manual searches in Google by two independent searchers yielded result publications for another 27% of trials. The manual search, therefore, increased the overall proportion of result publications to 75% for the three academic institutions.

Finally, some private drug and medical device companies operate company-specific databases that might list publications from completed trials. In response to results from TrialsTracker, a blogger, Adam Jacobs, argued that the 45% of trials that TrialsTracker found to be undisclosed shrink to 21% if one searches in these company-specific databases (see The Stats Guy blog) ^33^. However, each of these company-owned databases functions in a different way, and many companies do not publish such databases.

### 10. Inter-rater reliability

Expertise, ideally from more than one rater, is required to determine whether a publication matches a specific trial, and can thus be considered ‘published’. This certainly applies if manual searches in databases or internet search engines are added to automated registry and database checks. In Kasenda *et al*., for example, two investigators working independently, and in duplicate determined whether identified publications matched the corresponding protocol ^12^.

As we also applied manual searches in our explorative follow-up study, we had at least two researchers independently search for publications of registered trials. Although all researchers had a background in systematic review methodology and were trained in identifying result publications for clinical trials, we faced high inter-rater differences. For 16% of all trials, a publication was found by only one person, and for 10% of all trials, two different publications were found.

## Discussion

In this paper, we identified and characterized 10 variables influencing the tracking of whether and how timely clinical studies from academic institutions and private companies are disseminated. We further demonstrated the current opportunities and challenges of using these variables in tracking studies. Some of these variables need further conceptual and normative clarification.

**Responsible party for clinical studies**: First, we see the need to revisit our understanding of who is “responsible” for the timely (and unbiased) publication of trial results. All physicians functioning as investigators and their universities should not feel responsible only for trials where they are the legally defined “responsible party”. Investigators (both principal and co-investigators) and the hosting academic institutions are ethically obliged to proactively force the timely publication of all trials that recruited “their” patients, even if they only recruited as a cooperating partner. The risks and burdens for patients participating in clinical trials are justifiable only if the study generates social value in terms of knowledge gains. Therefore, the timely and unbiased publication of trial results is to be understood as a basic promise each physician investigator gives to participants recruited for a specific study.

Current efforts to benchmark universities should consider the current legal and ethical perspectives on “responsibility” for timely publication. This requires following up on *all* clinical studies that recruit patients from a given UMC, irrespective of whether the university was the sponsor/principal investigator or only a cooperating partner/facility. Data on the publication ratios of the two samples could be reported separately. 

**Types and characteristics of clinical studies:** Observational clinical studies are less regulated than interventional drug and device trials, and they do not face mandatory registration or reporting policies. From an ethical and economic perspective, however, those conducting observational studies have the same duties to increase value and reduce waste in biomedical research. For pragmatic reasons, follow-up studies currently focus on clinical studies that face mandatory registration and reporting, but future activities should also aim to shed more light on the registration and publication practices for other types of clinical studies.

**Status of clinical studies:** Reporting on the results or relevant barriers of discontinued, withdrawn, or early terminated studies is governed by the same ethical guidelines as completed clinical studies. Reporting does not necessarily require peer-reviewed publications but could also include reporting at registry websites and data-sharing platforms. The reporting of discontinued trials is a relevant measure to benchmark universities’ and companies’ contibutions to increasing value and reducing waste in research. The extent of trial discontinuation itself, however, does not serve as an appropriate measure for benchmarking activities. Academic institutions or companies conducting many complex or high-risk trials where discontinuation might be more probable than in simple trial designs should not be censured or discouraged. Furthermore, stigmatizing the discontinuation of trials might result in the inappropriate continuation of trials.

**Sources for sampling:** As long as study registration is not mandatory for all clinical studies, follow-up studies sampling at the registry level will most likely overestimate the true proportion of published clinical studies. To better understand the reporting performance of individual academic institutions and companies for non-registered clinical studies, follow-up studies must sample at the IRB level. Another way to improve opportunities to evaluate the reporting of, for example, observational studies would be legal or institutional policies requiring the prospective registration of all clinical studies.

**Timing of registration:** Little is known about how much the timing of registration affects the likelihood of results publication of clinical studies. Our above-reported results from a pilot study indicate a higher proportion of results publication in retrospectively registered trials. Follow-up studies interested in benchmarking the timely publication of trial results for universities or companies, therefore, should either focus on samples of prospectively registered trials to avoid bias or at least report the subgroup results for all prospectively and retrospectively registered trials.

**Determination of completion date:** Another normative and policy-oriented question is whether the appropriate definition of “completion” should refer to the PCD or the CD (see definitions above). Should certain trials be labeled as “not timely published” with reference to a 24-month time window after the PCD, even if they published the primary and secondary outcomes within 24 months after the CD? In line with European law (536/2014 Art 37.4), we propose taking the CD as the start date when following up on the reporting of summary results and peer-reviewed publications.

**Timeliness of dissemination:** We need a standard for what counts as “timely” publication. Several laws require the publication of summary results for certain types of studies within 12 months after the PCD (USA) or the CD (EU). Many other national laws, however, have no requirement in this regard. What should be seen as an ethically justifiable time to appropriately disseminate trial results via peer-reviewed publications or other dissemination formats? We propose 24 months after the CD as a potential normative standard for “timely publication” of peer-reviewed publications or similarly comprehensive and contextualized dissemination formats. Even for busy researchers, this 24-month period should allow enough time to publish. Publishing peer-reviewed results more than 24 months after the CD is, of course, better than not reporting them at all, but it should not be labeled as being “timely”. According to recent follow-up studies, less than 15% of all trials reported summary results within 12 months after the PCD ^14^, and less than 30% produced peer-reviewed publications within 24 months after the CD ^6^.

**Format of publication/dissemination**: Another complex issue is what types of publication or dissemination of trial results one should accept as appropriate. Most follow-up studies currently search for published summary results and/or peer-reviewed publications. But what about trial results provided via data-sharing platforms only or by industry-owned databases? Recent follow-up studies have demonstrated that accepting other publication formats, such as CSRs, yields higher publication rates ^8^. It is problematic, however, that these publications are not more easily accessible. Sponsors or investigators should directly link all publications. irrespective of their publication format, to the relevant registry entry of the respective trial. Registries can thus become the one-stop shop for clinical trial stakeholders (e.g., physicians, patients, systematic review and clinical guideline groups, meta-research, oversight).

**Sources for tracking:** As indicated above, at present, the addition of manual searches for publications in internet search engines and for CSRs in industry-owned trial databases yields a much higher number of result publications. Follow-up studies should explicitly acknowledge these limitations if they apply less extensive searches. From a normative perspective, however, these limitations and complexities in searching for result publications are themselves a problem. The above-mentioned registry entry as a one-stop shop for clinical trial results could solve this problem. Academic institutions and private companies should develop policies and incentives for linking result publications with the registry entry of the relevant trial.

**Inter-rater reliability:** Our finding that even experts trained in searching for clinical trials relatively often found different publications for the same trials once again speaks in favour of the one-stop shop approach.

Our study has the following limitations. First, although we reviewed more than 30 follow-up studies, and engaged in following up on clinical trials from German UMC, we might either have missed important tracking variables, or framed the 10 outlined tracking variables in a way that underplays certain aspects that deserve more attention. Second, in our outlines and the discussion of the 10 tracking variables, we only gave examples of how they are currently operationalized in follow-up studies. We will publish more detailed descriptions of the existing body of follow-up studies elsewhere.

## Recommendations

First, registration and publication activities for specific trials should be better linked — from publication to registry entry and from registry entry to publication. This applies not only to peer-reviewed papers including the NCT but also to CSRs, PhD theses, and other formats for results publication. Consequently, registry entries for clinical studies should become a one-stop shop for clinical study stakeholders. If successful and broadly established, the threaded publications initiative might become an even more appropriate one-stop shop in this regard ^34, 35^.

Second, to reach this goal, academic institutions and private companies should develop policies and incentives to further improve i) the registration of all their clinical studies, ii) the timely publication of all their completed and discontinued studies, and iii) the effective linkage of registry entries and their respective publications.

Third, future follow-up and tracking studies, as well as consequent rankings/benchmarking for academic institutions and private companies, should be transparent on how they specified the 10 tracking variables outlined in this paper and why they did so with regard to their follow-up objectives.

Recent efforts at more systematic, comprehensive, and sustained follow-up/tracking activities around the timely publication of clinical studies in combination with recent announcements from leading funders to require and evaluate result publication suggest an intensified public interest in the topic. Therefore, ranking and benchmarking academic institutions and private companies according to their registration and publication efforts seems to be a logical consequence. We hope that our clarification of relevant tracking variables will help make these new assessment activities more valid, effective, and efficient from the start. 

## Software availability

The R script used to as part of this study is available from Open Science Framework https://doi.org/10.17605/OSF.IO/FH426 ^15^

Licence: MIT License

## Data availability

All data underlying the results are available as part of the article and no additional source data are required.

Data generated in the pilot study as well as the subsequent follow-up study of all German UMCs will be published in a separate publication that will be linked in the Open Science Framework registration of the project.

OSF: Dataset 1: IntoValue. https://doi.org/10.17605/OSF.IO/FH426 ^15^

The data is available under a CC0 1.0 Licence

## Extended data

The methods for our explorative follow-up study have been published as a preregistered study protocol, available from Open Science Framework.

OSF: Extended data: IntoValue. https://doi.org/10.17605/OSF.IO/FH426 ^15^

Available under a CC0 1.0 licence
