# Supplementary material for: Tracking the timely dissemination of clinical studies. Characteristics and impact of 10 tracking variables
Source: F1000Res. 2018 Nov 29;7:1863. [Version 1] doi: 10.12688/f1000research.17022.1 (PMC6518431; doi:10.12688/f1000research.17022.1)
Supplement: Peer review history

# Peer review: Reviewer response for version 1

**DOI:** 10.5256/f1000research.18611.r47054

**Published:** 2019-05-13

**Authors:** Rabia Bashir

**Affiliations:** 1 Centre for Health Informatics, Australian Institute of Health Innovation, Macquarie University, Sydney, NSW, Australia

This manuscript presents a study based on 10 variables influencing the tracking of clinical studies and timely dissemination of their published results. The selection of these tracking variables is driven by expert knowledge and discussion among authors. To validate the completeness of these tracking variables, the authors used a sample of 34 studies that followed up on the publication of registered clinical trials of three leading German university medical centres (UMC). Based on a review of these studies, the authors make several recommendations. These include strengthening links between registry entry and publications and coordination among academic institutions and private companies to ensure the registration of clinical studies.

 The study addresses an interesting topic by listing a range of factors that influence the tracking and dissemination of clinical evidence. The paper is generally well written and structured. It would be interesting to see the detailed results; which the authors are aiming to publish elsewhere.

 I liked the study idea and agree with most of the conclusions that the authors have drawn and the recommendations that the authors have made. I agree that observational studies need to be registered because they can be used to influence policy and provide basis for clinical decision making. Also, the finding that up to 75% of published results can be linked to registry entries by adding manual searches support the conclusions made in another systematic review (Bashir *et al.*, 2017 ^1^).

 However, I have a few minor suggestions for strengthening the research:

- Studies are likely to be a biased representation because they were selected from leading German university medical centres for selecting the studies. They find that 35% are published at 24 months after completion date. It is not clear how these results might generalise so it is important that the conclusions do not over-generalise.
- The standard for “timely publication” was defined to be 24 months after the completion date. Using this as a standard for what counts as publication is arbitrary and while there are some details on mandates and policies the standard could have been better justified by the authors. A possible alternative might be to consider a model that estimates the median time to publication using a range of characteristics as factors, and avoid needing to choose a specific amount of time to judge what is timely and what is not.
- I agree with the authors’ recommendation that academic institutions and private companies need to play a role to improve the registration of clinical trials and their links with published results. However, journals are also often responsible for ensuring that trial registration information is provided. First, by following ICMJE guidelines on prospective registration. Second, they can ensure the identifier is included in the abstract and metadata they send to bibliographic databases. This will help to ensure that journals do not publish any clinical trials where trial identifier is not included in paper by authors or trial investigators.
- The manuscript can get benefit from these references: Trinquart *et al.* (2018 ^2^) and Bashir *et al.* (2017 ^1^).

I have read this submission. I believe that I have an appropriate level of expertise to confirm that it is of an acceptable scientific standard.

## References

[1] BashirRBourgeoisFDunnA: A systematic review of the processes used to link clinical trial registrations to their published results. *Systematic Reviews*.2017;6(1) : 10.1186/s13643-017-0518-3 10.1186/s13643-017-0518-3 28669351PMC5494826

[2] TrinquartLDunnABourgeoisF: Registration of published randomized trials: a systematic review and meta-analysis. *BMC Medicine*.2018;16(1) : 10.1186/s12916-018-1168-6 10.1186/s12916-018-1168-6 30322399PMC6190546

# Peer review: Reviewer response for version 1

**DOI:** 10.5256/f1000research.18611.r47315

**Published:** 2019-05-10

**Authors:** Shelly Pranić

**Affiliations:** 1 Department of Public Health, School of Medicine, University of Split, Split, Croatia

I agree with Reviewer 1 in that this study by Strech *et al.* provides a comprehensive list of variables to take into account when tracking the publication of clinical trial data. Now, with their applicable checklist, researchers can fine-tune their tracking of the data from trials. The paper is well-written, cites relevant literature, and describes the chosen variables well; however, since a part of this research involves a qualitative portion, it would be informative to describe the 'explorative review' clearer. Additionally, point 7 could be expanded. Please find comments below for your review, thank you.

 Methods:

1. Regarding the 'explorative review,' it would be interesting to know what was the initial list of tracking variables compiled by the authors before arriving at 10. The cited methodology on OSF does not describe this step. It is unclear how the authors voted or decided on the tracking variables. This explorative review is qualitative in nature and the authors should describe the way they chose the variables with greater clarity so that this part of the study could be replicated by other researchers.

Discussion:

1. Timeliness of the publication: I agree that researchers have limited time to publish, but as these tracking variables are also intended for assessing timeliness of companies' publications (including pharmaceuticals), the authors could also mention that there could be embargoes set forth by pharmaceutical industry officials and how embargoes could play a role in the timeliness of the publication of trial data irrespective of authors' available time to publish. I think this is a point worth mentioning in this section of the Discussion.

I have read this submission. I believe that I have an appropriate level of expertise to confirm that it is of an acceptable scientific standard.

## References

[1] Pauli-MagnusC: Referee Report For: Tracking the timely dissemination of clinical studies. Characteristics and impact of 10 tracking variables [version 1]. *F1000Research*.2019;7(1863) : Reference source 10.12688/f1000research.17022.1PMC651843131131084

# Peer review: Reviewer response for version 1

**DOI:** 10.5256/f1000research.18611.r44341

**Published:** 2019-02-21

**Authors:** Christiane Pauli-Magnus

**Affiliations:** 1 Department of Clinical Research, University of Basel , Basel, Switzerland

The paper by Strech *et al.* on tracking the timely dissemination and publication of clinical study results addresses a very relevant topic especially in academic clinical research. Since the metrics on published trials per institution/organisation are easily available through public databases such as the ‘trialstracker’ project, the ethical and scientific dimension of incomplete publication of clinical study results is gaining increasing public attention. Consequently, universities and university hospitals are asked to define measures on how to track and improve publication rates.

 Based on a literature review and an exploratory tracking of registered clinical trials conducted at three leading German university medical centres, the authors examine and discuss ten different tracking variables for clinical study publications. They finally come up with a set of recommendations on how academic institutions and private companies could increase trial publication.

 In my opinion, the work is clearly presented and cites the relevant literature. It uses appropriate methods and gives sufficient detail to be replicated by others. However, the detailed results of the review of follow-up studies is not given in the paper as it is planned to be published elsewhere.

 While I agree with the overall methodological approach and the results, I do not agree with the entire conclusion:

1. Responsible party for clinical studies: according to ICH-GCP, the overall responsibility for a clinical study including the publication lies with the study sponsor. Institutional follow-up on studies where the overall responsibility is with a third party (e.g. with a commercial sponsor or another academic institution in case of academic multicentre trials) will increase the bureaucratic burden of the institution without increasing publication rates. I would rather enforce that academic institutions establish policies to follow-up on the trials where they have sponsor-investigator responsibilities.
2. Source of sampling: Sampling studies at the IRB level will not resolve the problem of incomplete/inaccurate sampling. For instance, a positive IRB vote does not necessarily mean that a study has been started and IRB data on study discontinuation are probably as incomplete as registration data in public registries. Furthermore, depending on national legislation, IRB data are not publically accessible. I therefore would strongly support to rather invest in institutional policies that support mandatory study registration in a public study registry.
3. Timeliness of dissemination: I disagree that a 24-month period after CD should be enough time to publish even for busy investigators. This might be the case for studies, which are rapidly accepted for publication. In the case of studies with e.g. negative results, or results from discontinued/incomplete studies, substantial delay can derive from submission and resubmission processes. The clear definition of ‘timely’ therefore needs a publication platform that is equally accessible to all study sponsors, independent of study ‘success’.

On the other hand, I strongly support the overall recommendations that registration and publications activities should be better linked. I also think that registration and publication should be tracked for all prospective interventional AND observational clinical research and should include publication tracking for discontinued/incomplete studies. I like the idea that academic institutions should develop policies and incentives to improve registration and publication. By supporting clinical researchers during registration as part of a mandatory policy established at the University Hospital Basel in 2018, we could double the number of studies registered at clinicaltriasl.gov within one year. These are relatively easy measures with substantial local impact. However, on a national/international scale I am convinced that only a multi-stakeholder approach, including e.g. academic institutions, funders, IRBs and publishers will improve traceability and publication of study results.

 .

I have read this submission. I believe that I have an appropriate level of expertise to confirm that it is of an acceptable scientific standard.
